# Protein Quality Control in the Nucleus

**DOI:** 10.3390/biom4030646

**Published:** 2014-07-09

**Authors:** Sofie V. Nielsen, Esben G. Poulsen, Caio A. Rebula, Rasmus Hartmann-Petersen

**Affiliations:** Department of Biology, University of Copenhagen, Ole Maaløes Vej 5, DK-2200 Copenhagen N, Denmark; E-Mails: svnielsen@bio.ku.dk (S.V.N.); egpoulsen@bio.ku.dk (E.G.P.); caio.antonio@bio.ku.dk (C.A.R.)

**Keywords:** ubiquitin, degradation, chaperone, proteasome, misfolding, stress, SUMO

## Abstract

In their natural environment, cells are regularly exposed to various stress conditions that may lead to protein misfolding, but also in the absence of stress, misfolded proteins occur as the result of mutations or failures during protein synthesis. Since such partially denatured proteins are prone to aggregate, cells have evolved several elaborate quality control systems to deal with these potentially toxic proteins. First, various molecular chaperones will seize the misfolded protein and either attempt to refold the protein or target it for degradation via the ubiquitin-proteasome system. The degradation of misfolded proteins is clearly compartmentalized, so unique degradation pathways exist for misfolded proteins depending on whether their subcellular localization is ER/secretory, mitochondrial, cytosolic or nuclear. Recent studies, mainly in yeast, have shown that the nucleus appears to be particularly active in protein quality control. Thus, specific ubiquitin-protein ligases located in the nucleus, target not only misfolded nuclear proteins, but also various misfolded cytosolic proteins which are transported to the nucleus prior to their degradation. In comparison, much less is known about these mechanisms in mammalian cells. Here we highlight recent advances in our understanding of nuclear protein quality control, in particular regarding substrate recognition and proteasomal degradation.

## 1. Introduction

As a result of cellular stress, genomic mutations or defects in transcription, translation, intracellular trafficking or association with other macromolecules, cells continuously produce misfolded proteins. All cells have therefore evolved various measures to cope with the presence of such partially denatured proteins. The two main alleviatory strategies employed are either to shield the misfolded proteins from aggregation and refold them to the native state, or, if the native state is unachievable, to target the clients for degradation [1,2,3,4,5]. Faults in either of these systems lead to accumulation of toxic protein species which in turn may trigger diseases, including several neurodegenerative disorders [6]. Presumably to avoid the hazard of misfolded proteins forming intracellular aggregates, the cellular quality control systems appear to follow a better-safe-than-sorry principle, and are thus prone to also target proteins which are structurally perturbed, but still functional. An example of this is in cystic fibrosis, where degradation of certain mutant, but functional, variants of the chloride channel, CFTR, leads to lowered steady-state amounts of the channel protein which in turn leads to manifestation of the disease [7,8]. It therefore becomes clear that substrate selection is a tradeoff to allow for both recognition of a wide variety of misfolded substrates and a high specificity.

In eukaryotic cells most proteins are degraded via the ubiquitin-proteasome system (UPS) [9]. In this pathway, the specificity is ensured by labeling substrates with a chain of ubiquitin moieties. This polyubiquitylation process requires the sequential action of three types of enzymes: E1, E2, and E3. First, ubiquitin is bound and activated by the E1 ubiquitin-activating enzyme in an ATP-dependent reaction. Then the activated ubiquitin moiety is transferred to the E2 ubiquitin-conjugating enzyme which finally, via the E3 ubiquitin-protein ligase, transfers ubiquitin to the target protein. The human genome encodes two ubiquitin-specific E1s, more than 30 E2s [10] and more than 600 E3s [11]. Accordingly, the specificity in degradation lies with the substrate-binding E3s. Thus, E3s specific for misfolded proteins must be able to recognize some structural aspect which is common in a variety of misfolded proteins, but absent in their native counterparts. The distinguishing feature is generally believed to be the exposure of hydrophobic areas. However, as of yet, only the yeast E3 San1 has been shown to directly recognize misfolded proteins [12]. The other E3s involved in degradation of misfolded proteins appear to have outsourced the substrate recognition to molecular chaperones [13]. For instance, the E3 CHIP binds directly to Hsp70-type chaperones and co-chaperones to ubiquitylate their clients [3,14]. Hence, chaperones are not solely protein folding factors, but also facilitate sorting of non-native proteins for degradation. Chaperones constantly inspect the cell for non-native proteins, and upon encountering a misfolded protein, they prevent aggregation and promote protein folding and degradation. When chaperones support protein degradation, this can occur either by the chaperone acting as a substrate recognition factor for an E3, and/or simply by maintaining the substrate in a soluble form to allow the E3 direct access. Clearly this is particularly important during cell stress, but since certain chaperones in the absence of clients are themselves targeted for degradation, this mechanism also ensures that induced chaperone levels are returned to basal levels after a period of stress [15].

Although the substrate specificity in general lies with the E3s, some level of specificity is also provided downstream of the E3-catalyzed ubiquitylation [16]. Thus, insoluble ubiquitylated proteins are targeted to the proteasome by the abundant p97/Cdc48 ATPase complex [17] and other chaperones. At the 26S proteasome, co-chaperones such as BAG-1 ensure docking of Hsp70-bound clients [3]. Presumably this tight chain of custody ensures that non-native proteins are constantly kept in check until they are degraded. Finally, at the 26S proteasome the degradation of misfolded proteins is further ensured by Hul5 [18,19], a proteasome-associated E3, and certain deubiquitylating enzymes (DUBs) such as Rpn11, Uch37, Ubp3 and Ubp6, that may either promote degradation or function as a final failsafe mechanism to counter degradation [20,21,22,23,24].

**Figure 1:**
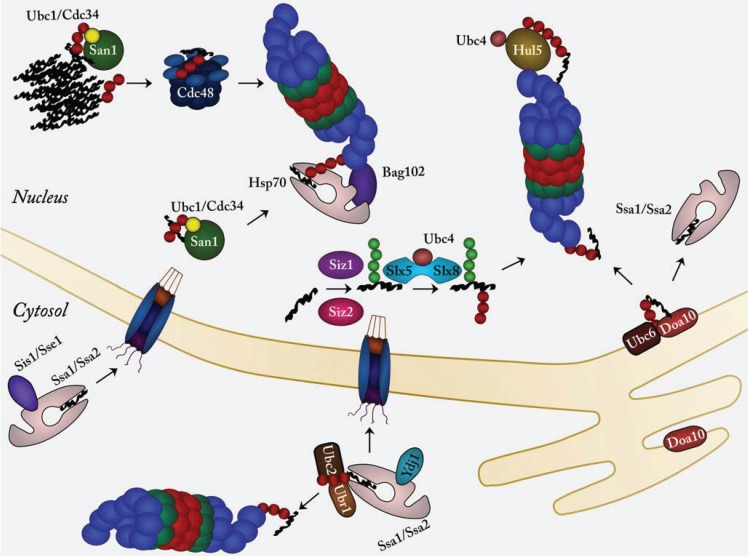
Pathways for nuclear protein quality control in yeast. Conjugating ubiquitin (red sphere) to misfolded proteins (black thread) occurs in the nucleus primarily via the San1 E3 ubiquitin-protein ligase (green sphere) and its cognate E2 enzymes Ubc1 (yellow) and Cdc34 (dark red). In fission yeast the Bag102 Hsp70/proteasome co-factor links Hsp70 chaperones (pink) directly with the 26S proteasome. The transmembrane ER/nuclear envelope E3 ligase Doa10 (red) and its cognate E2 Ubc6 (brown) also target certain Ssa1 and Ssa2 Hsp70 (pink) clients for proteasomal degradation. The Slx5-Slx8 heterodimeric STUbL (blue) targets misfolded proteins that have first been marked with SUMO (green sphere) by the SUMO E3s Siz1 and Siz2. The Sis1 and Sse1 co-chaperones (purple) mediate transport of cytosolic chaperone clients to the nucleus. Due to some substrate overlap between San1 and the cytosolic Ubr1 E3 ubiquitin protein ligase, Ubr1 (light brown) and its cognate E2 Ubc2 (dark brown) may also participate in nuclear protein quality control. Finally, at the 26S proteasome the proteasome-associated E3 Hul5 (yellow), may further ubiquitylate the misfolded substrates, to ensure efficient degradation. See text for further details.

As protein misfolding may occur in any cellular location, cells must also have protein quality control measures in place to target any misfolded protein, regardless of its subcellular location, for degradation [25,26].

Thus, the ER-associated degradation (ERAD) pathway targets misfolded ER/secretory proteins, and mitochondria are also equipped with specialized protein quality control systems. The cytosolic and nuclear protein quality systems are somewhat interlinked, but some clear distinctions are seen. For instance, some quality control relevant E3s are exclusively located in the nucleus. Finally, data, primarily from yeast, suggest that certain misfolded cytosolic proteins are transported to the nucleus for degradation [27] (Figure 1). Whether this is also a common mechanism in mammals is still not clear. Yeast cells and other unicellular eukaryotes undergo closed mitosis, where the mother nucleus is not dismantled during cell division. This ensures that the nucleo-cytoplasmic compartmentalization is never lost and may make the nucleus an ideal environment for degradation of misfolded proteins. In addition, the subcellular localization of proteasomes differs between yeasts and mammals. In mammalian cells proteasomes are evenly spread throughout the cytosol and nucleus [28], but in yeast most proteasomes are located along the inside of the nuclear envelope [29]. Hence, the yeast nucleus is likely to be more active in protein degradation. These subtle distinctions could make yeast cells more dependent on a pathway involving nuclear import. In the following we will review protein quality control, E3s (Table 1) and degradation mechanisms in the nucleus.

**Table 1:** E3 ubiquitin-protein ligases in nuclear protein quality control.

Budding Yeast	Human
San1	−
Ubr1	UBR1/N-recognin 1
Ubr2	UBR2/N-recognin 2
Doa10	TEB4
Slx5-Slx8	RNF4
Hul5	UBE3
−	Arkadia/RNF111
−	E6-AP
−	UHRF2
−	CHIP

## 2. Nuclear Protein Quality Control

### 2.1. The E3 Ubiquitin-Protein Ligase CHIP Targets Chaperone Clients for Degradation

One of the best characterized E3s involved in targeting misfolded proteins for degradation is mammalian CHIP. Through tetratricopeptide repeats, CHIP directly interacts with Hsp70s and Hsp90 to ubiquitylate the associated clients [14]. Studies have linked CHIP to the degradation of a broad range of chaperone clients, including signaling proteins such as Raf-1, steroid hormone receptors and p53, which associate with chaperones as a regulatory mechanism during signal transduction. However, as an E3 specific for chaperone client proteins, CHIP substrates also include misfolded and aggregation prone proteins such as mutant versions of SOD-1 and hyperphosphorylated Tau that are recognized by chaperones during protein quality control [14].

As an E3 directly associated with chaperones and targeting their clients, CHIP is not unique. The E6-AP E3 ligase, which is only found in higher eukaryotes, also interacts with Hsp70-type chaperones and promotes degradation of bound substrates [30].

Presumably since chaperone clients are not all irreversibly misfolded, CHIP mediated degradation is highly regulated by various co-chaperones. Upon binding of BAG-1 and HSJ-1, the degradation of CHIP clients is enhanced. HSJ-1 is a member of the J-domain family of Hsp70 client-loading factors, but also contains ubiquitin interaction motifs. This combination of domains enables HSJ-1 to bind ubiquitylated clients after CHIP-catalyzed ubiquitylation [31], thus ensuring binding of the ubiquitylated cargo to Hsp70 en route to the proteasome. At the 26S proteasome the other co-chaperone, BAG-1, may then take over. BAG-1 contains an N-terminal UBL domain that interacts with the 26S proteasome and a C-terminal BAG domain that binds Hsp70 [32,33,34]. This allows BAG-1 to facilitate docking of the client-loaded complex at the proteasome. The BAG-1 binding site on the 26S proteasome has yet to be determined, but is likely to overlap with the binding sites for other UBL domain proteasomal co-factors such as Rad23/HHR23 and Dsk2/PLIC [35].

In addition to positive regulators, CHIP activity can also be attenuated. Co-chaperones that promote folding pathways compete with HSJ-1 and BAG-1. For instance, Hsp70 and Hsp90 bind in a mutually exclusive manner to CHIP, while the Hsp70 interacting protein HIP competes with BAG-1 in binding to the ATPase domain of Hsp70 [36]. In addition the Hsp70 co-chaperones BAG-2 and HspBP1 both function to inhibit degradation [4,14,37]. Upon binding, they inhibit the ubiquitin ligase activity of CHIP in chaperone/co-chaperone complexes, thus allowing chaperone clients an opportunity to fold [4,38].

As for misfolded nuclear CHIP substrates, not much is known. However, since CHIP targets signaling proteins located in the nucleus (e.g., steroid hormone receptors and p53), it is likely to also be involved in targeting misfolded nuclear proteins for degradation. Although no CHIP orthologue is found in yeast, fission yeast encodes two orthologues of human BAG-1 called Bag101 and Bag102. While Bag101 was shown to regulate turnover of native Rad22, a protein involved in homologous recombination [39], Bag102 was shown, similar to BAG-1, to co-ordinate the transfer of a mutant, and presumably structurally perturbed, kinetochore protein to the 26S proteasome [34]. When performing this function, Bag102 is located in the ER/nuclear membrane, presumably like 26S proteasomes along the inside of the nuclear envelope [29]. Thus, since at least some functions of BAG-1 are conserved to species that lack CHIP orthologues, other E3s must function like CHIP in conjunction with Hsp70, and perhaps Hsp90. This is further supported by the observation that chaperone-assisted degradation is not strongly affected in CHIP-deficient mammalian cells [40], while targeted disruption of BAG-1 in mice leads to early embryonic lethality [41].

### 2.2. San1, an Intrinsically Disordered E3 Ubiquitin-Protein Ligase Specific for Misfolded Proteins

The connection of San1 with nuclear protein quality control was made through the observation that budding yeast *san1* mutants were isolated as suppressors of both the *sir4-9* and *cdc68-1* temperature sensitive mutants [42]. This suggested that perhaps at the restrictive temperature, the mutant but functional, nuclear Sir4-9 and Cdc68-1 proteins were identified by a nuclear protein quality control network as being misfolded, and subsequently degraded. Blocking this untimely degradation by compromising San1 activity would then restore protein levels and alleviate the mutant phenotypes. Accordingly, degradation assays confirmed a San1-dependent degradation of the Sir4-9 and Cdc68-1 proteins [42]. In general, San1 appears highly specific for aberrant proteins, as it does not target the wild-type versions of its mutant substrates.

In the degradation of mutant nuclear proteins, budding yeast San1 functions in conjunction with the E2s Ubc1 and Cdc34 [42], while fission yeast San1 instead relies on Ubc4 and Ubc5 [34,43]. San1 contains an alternate version of the canonical RING-domain common among RING-domain ubiquitin-protein ligases. In the RING domain of San1 the fifth zinc binding His/Cys residue is replaced by a glycine, which curiously does not render the E3 ligase inactive.

As proven by elaborate yeast two-hybrid screening and co-precipitation experiments, San1 so far appears to be unique in its ability to directly target misfolded proteins for degradation, and thus does not rely on substrate recognition by molecular chaperones [12]. Structure prediction algorithms assigned an intrinsically disordered structure to the areas of San1 flanking the RING domain. This prediction was confirmed biochemically via gel filtration, limited proteolysis and CD spectrometry [12]. Scattered across these disordered areas are several short hydrophobic stretches which act as substrate recognition sites. By introducing mutations in all potential binding sites and then mapping the interaction to known substrates, it was shown that no specific patch or patches were ubiquitously required for binding, but rather, the required binding profile varied with the substrate [12]. The varied distribution of the binding patches, combined with the high flexibility of the disordered regions, convey a vast diversity in the substrate recognition profile for San1. In this unique substrate recognition mechanism dubbed “disorder targeting misorder”, the conformational plasticity of San1 allows for the binding to each of the distinctly shaped, yet collectively misfolded proteins, and thus bypassing the need for chaperones (Figure 1). This hypothesis of intrinsic disorder as a means to convey flexible interaction patterns is also described for some chaperones and small heat-shock proteins [44,45,46]. It is interesting to note that, due to its innate ability to ubiquitylate a great variety of misfolded and unstructured proteins, San1 has been under selective pressure to not include any lysine residues in its otherwise mostly random N- and C-terminal sequences. The addition of a single lysine residue in these regions leads to cis-autoubiquitylation and rapid degradation [47]. Recent work on San1 mediated protein quality control has found an interesting link to the segregase function of Cdc48 [17]. It has been shown in *S. cerevisiae* that Cdc48 is not required for degradation of all San1 substrates, but that the requirement for Cdc48 is correlated to the insolubility of the San1 substrate (Figure 1). When the San1 substrates are most insoluble, the need for Cdc48 in their degradation is highest, and *vice versa*. Additionally it was shown that when Cdc48 activity is compromised, the substrate ubiquitylation levels increase, suggesting that San1 ubiquitylates the insoluble substrates prior to Cdc48-powered extraction [48].

Presumably due to its disordered nature and thus a low selection pressure to preserve structurally important residues, it has so far not been possible to identify any orthologue of San1 in higher eukaryotes. It is likely that a San1-like E3 exists in higher eukaryotes, but also possible that San1 function in mammalian cells is carried out by E3s such as CHIP, which are not found in yeast. In this respect, the human E3 UHRF-2 (also known as Np95, ICBP90-like RING finger protein or RNF107) which has been shown to target misfolded nuclear protein for degradation [49] is another possible candidate.

### 2.3. Hul5, a Proteasome-Associated E3 Ubiquitin-Protein Ligase

Once a misfolded ubiquitylated substrate docks at the 26S proteasome, it may become further ubiquitylated by the proteasome-associated E3 known as Hul5 in yeast (Figure 1) [19,50]. Unlike most other E3s, Hul5 generally targets proteins that are already ubiquitylated [51]. In this way Hul5 extends the ubiquitin chains and allows for a tighter and prolonged interaction with the 26S proteasome, thus accelerating degradation. Although Hul5 is unlikely to exclusively target misfolded proteins, Hul5 activity was shown to be especially pronounced following a heat shock [52]. Accordingly, several low-solubility proteins, including the prion-like protein Pin3 are Hul5 substrates [52].

Previously it was suggested that Hul5 and the deubiquitylating enzyme Ubp6 have antagonistic activities [19]. In accordance with this, compromising yeast Ubp6 and its mammalian homolog Usp14 were shown to accelerate the degradation of aberrant proteins [53,54]. Thus, Ubp6/Usp14 may provide proteins that are not terminally misfolded a last opportunity to escape.

Evidence suggests that Hul5 constantly shuffles between the cytosol and the nucleus. Interestingly, heat shock blocks Hul5 nuclear import [52], suggesting that Hul5 primarily functions in the degradation of misfolded cytosolic proteins. However, recently the fission yeast Hul5 orthologue was shown to contribute to the degradation of a misfolded nuclear kinetochore component [34], suggesting that at least in some cases, Hul5 is involved in nuclear protein quality control.

In comparison less is known about the human Hul5 orthologue, Ube3C. However, recently Ube3c was shown to partake in the degradation of proteins that are structurally perturbed [55]. However, from this study it was not clear if Hul5 functioned in the nucleus, cytosol or both.

### 2.4. Doa10, an ER/Nuclear Envelope Bound E3 Involved in Nuclear Protein Quality Control

Although the yeast E3, Doa10, is widely recognized for its role in the ER-associated degradation pathway [56,57] it also has the ability to ubiquitylate cytosolic and nuclear proteins and target them for proteasomal degradation [58,59]. Doa10 spans the membrane of the ER/nuclear envelope with its RING-domain facing the cytosol/nucleoplasm [58]. This localization allows for Doa10 to exert its role in protein quality control on both soluble proteins in the cytosol or nucleus (Figure 1) and proteins inserted in the membrane of the ER/nuclear envelope [59,60]. The mutant kinetochore protein Ndc10-2 is a substrate for Doa10-mediated protein quality control. In *doa10* null mutants, Ndc10-2 is completely stabilized which in turn alleviates the temperature sensitive growth-phenotype of the *ndc10-2* mutant. In addition to Doa10, the Hsp70-type chaperone Ssa1 and to a lesser extent the Hsp40-type co-chaperone Sis1, are required for Ndc10-2 degradation [61,62]. However, in this case it is not known if the chaperone functions as a substrate receptor for Doa10 or by simply maintaining the substrate in an accessible soluble form. In their study of Ndc10-2 degradation [61], Furth and colleagues identified a 6-residue hydrophobic segment in the outermost C-terminus of Ndc10-2 that is essential for its ubiquitylation by Doa10 and subsequent degradation. Given the obvious hydrophobicity of this degron, it is somewhat surprising that deletion of Doa10 alone can lead to complete stabilization of Ndc10-2, and that other quality control E3s such as San1 do not appear to functionally overlap with Doa10 in ubiquitylation of this particular substrate. The mammalian Doa10 orthologue TEB4 has been reported as a functional ER-membrane localized E3. However, it remains rather uncharacterized compared to its yeast counterpart and has not yet been shown to participate in nuclear quality control [63].

### 2.5. SUMO-Targeted E3 Ubiquitin-Protein Ligases in Nuclear Protein Quality Control

In both yeast and mammalian cells, modification with the ubiquitin-like modifier, SUMO, has also been linked to quality control and the degradation of aberrant proteins. In protein quality control, sumoylation can serve as a recruitment platform for SUMO-targeted ubiquitin-protein ligases (STUbLs) that subsequently ubiquitylate the sumoylated protein to facilitate its degradation by the proteasome [64]. Upon both proteasome inhibition and in response to heat shock, high-molecular weight SUMO conjugates accumulate, suggesting a general role for SUMO in protein quality control [65].

In yeast, the heterodimeric STUbL Slx5-Slx8 (Rfp1/2-Slx8 in fission yeast) is located in the nucleus [66,67] and responsible for the ubiquitin-proteasome mediated degradation of a number of sumoylated substrates. Indeed, high molecular weight SUMO-conjugates accumulate in *slx5*Δ and *slx8*Δ strains [68,69]. The role of the Slx5-Slx8 heterodimer in protein quality control was first established when the two genes were identified as suppressors of a temperature sensitive mutant in the DNA binding protein Mot1 [70]. At the restrictive temperature, the mutant Mot1 protein is highly unstable, but stabilized in *slx5* and *slx8* deletion strains [71]. Since the temperature sensitive phenotype of the *mot1* mutant is alleviated when the protein is stabilized, the mutant Mot1 protein is, at least to some extent, still functional also at the restrictive temperature [70]. The stabilization of mutant Mot1 was also observed in mutants lacking the SUMO-ligases Siz1 and Siz2 [71], revealing that sumoylation is required for degradation (Figure 1). The mammalian STUbL RNF4 is an orthologue of budding yeast Slx5 and fission yeast Rfp1/2, however, as opposed to the heterodimers that function in yeast, RNF4 acts as a homodimer that can interact with one E2 protein [72]. While the vast majority of RNF4 substrates are proteins that are degraded in response to a regulatory stimulus, the best studied RNF4 substrate is the abnormal oncoprotein PML-RARα, which drives the pathogenesis of the malignancy acute promyelytic leukemia. The normal PML protein constitutes the outer sphere of PML nuclear bodies (PML-NBs), which are highly heterogeneous and dynamic subnuclear structures [73]. Functionally, PML-NBs have been implicated as sites of regulation of apoptosis, cellular senescence, antiviral responses, inhibition of proliferation and maintenance of genomic stability [74]. The PML-RARα oncoprotein interferes with the formation of PML-NBs and thus diminishes the tumor suppressor activities that are usually present in these structures. Arsenic trioxide is one of the therapeutic agents used in the treatment of acute promyelytic leukemia [73]. RNF4 plays an important role in the molecular mechanism behind this treatment, as it targets PML-RARα for degradation in a SUMO-dependent manner [75,76]. The As_2_O_3_ induced oxidation of PML-RARα causes multimerization of PML-RARα proteins and at the same time it increases the affinity of PML-RARα for the SUMO-conjugating enzyme Ubc9 leading to PML-RARα sumoylation [77,78]. The sumoylation is recognized by the SUMO-interacting motifs of RNF4 and leads to RNF4-dependent polyubiquitylation of PML-RARα ultimately causing its degradation by the proteasome and reestablishment of PML-NBs [75,76]. Furthermore, the less characterized STUbL Arkadia/RNF111 is also able to degrade PML-RARα in a SUMO-dependent manner. However, this pathway seems to be different from the RNF4 pathway as simultaneous siRNA knockdown of both STUbLs, further increases the As_2_O_3_ induced sumoylation compared to the single knockdowns [79].

### 2.6. Ubr1 in Nuclear Protein Quality Control

In budding yeast, Ubr1 is a cytosolic E3 ubiquitin ligase, that along with its cognate E2, Ubc2, is reported to degrade substrates both of the N-end-rule pathway and in cellular protein quality control [80,81]. When Ubr1 exerts its role in protein quality control it does so in collaboration with the Hsp70-type chaperones Ssa1/Ssa2 (Figure 1) and the co-chaperones Sis1 and Ydj1 (Hsp40) and Sse1 (Hsp110) [82,83,84]. Unlike San1 [12] a specific substrate-recognition region in Ubr1 has not been mapped. However, since Ubr1-mediated ubiquitylation requires the chaperones Ssa1/Ssa2 and/or the co-chaperone Sse1 [82], it seems reasonable to assume that Ubr1 utilizes chaperones as substrate-recognizing entities. However, it is also possible that these chaperones stimulate Ubr1 ubiquitylation by keeping the substrates in a soluble state.

A role for Ubr1 in nuclear protein quality control has mainly been assigned because of the substrate overlap between Ubr1 and San1 [34,82,83,85]. Certain model substrates that are in fact cytosolic and can be ubiquitylated by Ubr1 accumulate in the nucleus when their degradation is inhibited and this accumulation seems to be dependent on the co-chaperone Sse1 [83]. The interaction between San1 and the cytosolic quality control model substrate NBD2* requires a functional Ssa1 [85], and the Hsp40 type II chaperone Sis1 is required for the transfer of yet another model substrate, CG*, into the nucleus where it is degraded in a San1 dependent manner [27]. It remains unknown what determines if the ubiquitylation is catalyzed by Ubr1 or San1, but the shuttling of substrates between the two E3s in different cellular compartments is performed by various chaperones and co-chaperones (see below).

The Ubr1 orthologue in *S. pombe*, Ubr11, is localized in the nucleus [86] and ubiquitylates the structurally perturbed kinetochore protein Spc7-23 to facilitate its degradation. Interestingly, San1 also ubiquitylates Spc7-23 indicating a substrate overlap between the two E3 ubiquitin ligases [34]. From these data it is not possible to determine if San1 and Ubr11 work in parallel or in the same pathway. However, based on the involvement of Hsp70-type chaperones and the co-chaperone Bag102 in Spc7-23 degradation, one could speculate that Spc7-23 could be degraded either by Ubr11 in collaboration with a Hsp70-Bag102 complex or in a chaperone-independent manner by San1 (Figure 1) [12]. The *S. cerevisiae* Ubr2 has also been implicated in protein quality control. However, this function has so far only been demonstrated for cytosolic substrates [87].

### 2.7. Misfolded Cytosolic Proteins Are Transported to the Nucleus for Degradation

Co-chaperones of the Hsp40 family have been shown to regulate the co-operation between the Hsp70 system and the UPS. In particular, the budding yeast Hsp40 Ydj1 was shown to suppress aggregation of short-lived GFP (slGFP), a terminally misfolded substrate for cytosolic chaperone-assisted degradation [84]. However, another Hsp40 co-chaperone, Sis1, was required for slGFP degradation [84]. In the degradation of slGFP, both the Ubr1 and San1 E3s were involved, suggesting that perhaps some slGFP substrate degradation occurs after San1 ubiquitylation in the nucleus. In support of this, Sis1 and its mammalian orthologue DnaJB1 were recently shown to be required for transporting misfolded cytosolic proteins to the nucleus for subsequent ubiquitylation and proteasomal degradation [27]. In these studies the model substrates were cytosolic misfolded carboxypeptidase Y and a mutant luciferase. The obtained data support a model where, at least in yeast, cytosolic misfolded protein is first recognized and kept in a soluble state by Hsp70-type chaperones and Ydj1. This is followed by ubiquitylation by Ubr1, and eventually recognition by Sis1 (Figure 1). The Sis1 interaction then mediates transport of the client protein to the nucleus. Inside the nucleus, San1 then further contributes to substrate ubiquitylation, before the substrate is finally degraded by the proteasome [27]. Interestingly, the presence of protein aggregates was shown to sequester Sis1, and thus inhibit degradation of the misfolded model substrate by impeding its nuclear import [27].

Previously, other reports have shown that misfolded cytosolic proteins in yeast are degraded in the nucleus [82,83]. Although at least part of this system is conserved to human cells whether misfolded cytosolic proteins are generally degraded in the nucleus remains to be shown. In mammalian cells proteasomes are fairly evenly distributed throughout the cytosol and nucleus [28], but in yeast most proteasomes (approximately 80%) are located in the nucleus [29]. This distinction could make yeast cells more dependent on a pathway involving nuclear import.

## 3. Concluding Remarks

In yeast, the significant contribution of nuclear protein quality control to overall cellular proteostasis is demonstrated by the fact that cytosolic quality control substrates are transferred to the nucleus for degradation. While it seems to be clear that this shuttling mechanism is mediated by chaperones and co-chaperones, the mechanism that decides whether a substrate remains in the cytosol or is transported to the nucleus, is still not clear. If the shuttling is merely a manifestation of an overflow of misfolded cytosolic substrates one would not expect an additive stabilizing effect in an *ubr1*Δ*san1*Δ strain, and this has indeed been shown for several substrates [82,83]. These observations support the requirement of a, yet unknown, regulatory mechanism that determines whether the protein is shuttled to the nucleus or stays in the cytosol.

In order to transfer the many important observations made in yeast regarding nuclear protein quality control, to a mammalian cellular system, it may be necessary to address the obvious difference in proteasome distribution between yeast and mammalian cells. San1 is one of the most thoroughly studied components of the yeast nuclear quality control system, yet no mammalian protein with structural and functional similarities to San1 has been identified. As extraordinary as San1 is in regard to combining the substrate recognition capabilities of a chaperone and ubiquitylation properties of an E3 in the same protein, it is possible that higher eukaryotes have evolved past this combined mechanism and instead rely solely on the two-step mechanism of chaperones as the substrate recognition factors that in turn recruit specialized E3s. In order to study the significance of nuclear protein quality control in higher eukaryotes and to identify the key E3s and chaperones involved, it may be necessary to broaden the scope and not solely focus on orthologues of yeast quality control components. To this end, it is important that mammalian nuclear quality control substrates are identified, and their degradation pathways are mapped. Finally, it is possible that nuclear protein quality control is further divided in various subnuclear compartments. Thus, perhaps the mechanisms and quality control requirements are different between e.g., chromatin, the nucleolus, and various nuclear bodies, such as PML-bodies.
